# Gender-Associated Genomic Differences in Colorectal Cancer: Clinical Insight from Feminization of Male Cancer Cells

**DOI:** 10.3390/ijms151017344

**Published:** 2014-09-29

**Authors:** Rola H. Ali, Makia J. Marafie, Milad S. Bitar, Fahad Al-Dousari, Samar Ismael, Hussain Bin Haider, Waleed Al-Ali, Sindhu P. Jacob, Fahd Al-Mulla

**Affiliations:** 1 Department of Pathology, Faculty of Medicine, Health Sciences Center, Kuwait University, P.O. Box 24923, Safat 13110, Kuwait; E-Mails: rolapath@hotmail.com (R.H.A.); hus4in@live.com (H.B.H.); waleed_mls@hotmail.com (W.A.-A.); sindhujacob@hsc.edu.kw (S.P.J.); 2 Kuwait Medical Genetics Center, Ministry of Health, Safat 13001, Kuwait; E-Mail: mj_marafie@yahoo.com; 3 Department of Pharmacology, Faculty of Medicine, Health Sciences Center, Kuwait University, P.O. Box 24923, Safat 13110, Kuwait; E-Mail: milad.bitar@gmail.com; 4 Department of Forensic Evidence, Ministry of Interior, P.O. Box 12500, Shamiya 71655, Kuwait; E-Mails: fahad77@hotmail.com (F.A.-D); sismaeel@yahoo.com (S.I.)

**Keywords:** X chromosome, comparative genomic hybridization, colorectal cancer, copy number aberration, gender

## Abstract

Gender-related differences in colorectal cancer (CRC) are not fully understood. Recent studies have shown that CRC arising in females are significantly associated with CpG island methylator phenotype (CIMP-high). Using array comparative genomic hybridization, we analyzed a cohort of 116 CRCs (57 males, 59 females) for chromosomal copy number aberrations (CNA) and found that CRC in females had significantly higher numbers of gains involving chromosome arms 1q21.2–q21.3, 4q13.2, 6p21.1 and 16p11.2 and copy number losses of chromosome arm 11q25 compared to males. Interestingly, a subset of male CRCs (46%) exhibited a “feminization” phenomenon in the form of gains of X chromosomes (or an arm of X) and/or losses of the Y chromosome. Feminization of cancer cells was significantly associated with microsatellite-stable CRCs (*p*-value 0.003) and wild-type *BRAF* gene status (*p*-value 0.009). No significant association with other clinicopathological parameters was identified including disease-free survival. In summary, our data show that some CNAs in CRC may be gender specific and that male cancers characterized by feminization may constitute a specific subset of CRCs that warrants further investigation.

## 1. Introduction

Genomic instability is an important molecular event in the development of colorectal cancer (CRC) [1], encompassing chromosomal instability, microsatellite instability and aberrant DNA methylation. Chromosomal instability is the most common type of genomic instability and is an early event in colorectal carcinogenesis [2] causing chromosomal copy number aberrations (CNA). CNAs in CRC have been extensively studied using cytogenetic techniques and comparative genomic hybridization (CGH) [3,4,5,6,7,8,9]. Yet, despite recent advances in CRC genomics, gender-specific CNAs have only been sporadically documented by CRC genomic studies. Excluding gender-specific cancers (e.g., prostate, breast), males and females in general differ in their susceptibility to different cancers and differ in clinical outcome, therefore, understanding gender differences is important in order to gain insight into disease biology and underlying pathogenic mechanisms [10]. Studying gender-related genomics can also help optimize therapeutic strategies particularly in the era of personalized medicine.

With respect to CRC, males and females show many epidemiological, clinical and pathological differences. It is plausible to hypothesize that at least some of these gender differences are related to genes encoded in the sex chromosomes. It has been previously demonstrated that X chromosome CNAs are frequent in CRC cells in both male and female patients [11,12]. However, a significantly higher frequency of these X chromosomal aberrations has been demonstrated in CRCs from male patients [6,13,14]. The clinical significance of this phenomenon is yet to be elucidated.

Are copy number aberrations in CRC gender-specific? To address this question, a cohort of 116 male and female patients with CRC was explored for chromosomal CNA by high-resolution array CGH (aCGH), with special attention to sex chromosomal aberrations. The aim was to specifically shed more light on the differences in CNAs between male and female colorectal cancer cells, and to correlate the aCGH findings with clinicopathological parameters, an aim that, to the best of our knowledge, has not been previously well-addressed.

## 2. Results

### 2.1. Demographic/Pathologic Data

Table 1 shows that of 116 patients included in this study, 59 (50.9%) were females and 57 (49.1%) were males. Mean age was 64.2 years (range from 35 to 94). Tumors were from different colonic sites with 28 tumors (24.2%) being right-sided (cecum to transverse colon), 47 tumors (40.5%) being left-sided (descending colon and sigmoid), 23 (19.8%) rectal, and 18 (15.5%) of unknown site. Histological differentiation was as follows: 13 tumors (11.2%) were well-differentiated, 84 (72.4%) were moderately-differentiated, 11 (9.5%) were poorly-differentiated, and in eight cases (6.9%) differentiation could not be determined. Most tumors (82.8%) in both male and female patients were Dukes’ stage B (84.2% in males and 81.4% in females). The majority of patients (75%) had microsatellite-stable cancers with wild-type *BRAF* gene (72% in males and 77.9% in females). Outcome data were available for 87 (75%) patients and the mean period of clinical follow-up was 7.5 years. In total, 63 patients (54.3%) were alive and disease-free 10 years after surgery, while 24 patients (20.7%) had disease relapse in the form of local recurrence or distant metastasis (Table 1). Female patients showed more recurrences than male patients (28.8% and 12.3%, respectively), but the disease-free survival did not significantly differ between the two genders (data not shown).

**Table 1:** Clinicopathological characteristics of the colorectal cancers classified based on gender.

Characteristics	Males No. (%)	Females No. (%)	Total No. (%)
No. Of Patients	57 (49.1)	59 (50.9)	116 (100)
Mean Age in Years	63	65.4	–
Age			
31–40 years	1 (1.8)	4 (6.8)	5 (4.3)
41–50 years	8 (14.0)	8 (13.6)	16 (13.8)
51–60 years	15 (26.3)	11 (18.6)	26 (22.4)
61–70 years	14 (24.5)	9 (15.2)	23 (19.8)
71–80 years	18 (31.6)	17 (28.8)	35 (30.2)
>81 years	1 (1.8)	10 (17.0)	11 (9.5)
Tumor Site			
Right side of colon	16 (28.1)	12 (20.3)	28 (24.2)
Left side of colon	18 (31.6)	29 (49.2) *	47 (40.5)
Rectum	11 (19.3)	12 (20.3)	23 (19.8)
Unspecified	12 (21.0)	6 (10.2)	18 (15.5)
Histological Grade			
Well-differentiated	4 (7.0)	9 (15.2)	13 (11.2)
Moderately-differentiated	44 (77.2)	40 (67.8)	84 (72.4)
Poorly-differentiated	6 (10.5)	5 (8.5)	11 (9.5)
Unspecified	3 (5.3)	5 (8.5)	8 (6.9)
Dukes’ Stage			
Stage B	48 (84.2)	48 (81.4)	96 (82.8)
Stage C	8 (14.0)	10 (16.9)	18 (15.5)
Stage D	1 (1.8)	1 (1.7)	2 (1.7)
Microsatellite Status—*BRAF*			
MSS—wild type *BRAF*	41 (72.0)	46 (77.9)	87 (75.0)
MSS—mutated *BRAF*	2 (3.5)	1 (1.7)	3 (2.6)
MSI—wild type *BRAF*	6 (10.5)	4 (6.8)	10 (8.6)
MSI—mutated *BRAF*	4 (7.0)	4 (6.8)	8 (6.9)
Unspecified	4 (7.0)	4 (6.8)	8 (6.9)
Follow-Up			
Relapse, local or metastatic	7 (12.3)	17 (28.8) **	24 (20.7)
Relapse-free	35 (61.4)	28 (47.5)	63 (54.3)
Unknown	15 (26.3)	14 (23.7)	29 (25.0)

* *p*-value = 0.040; ** *p*-value = 0.023.

### 2.2. Array Comparative Genomic Hybridization (aCGH)

**Figure 1:**
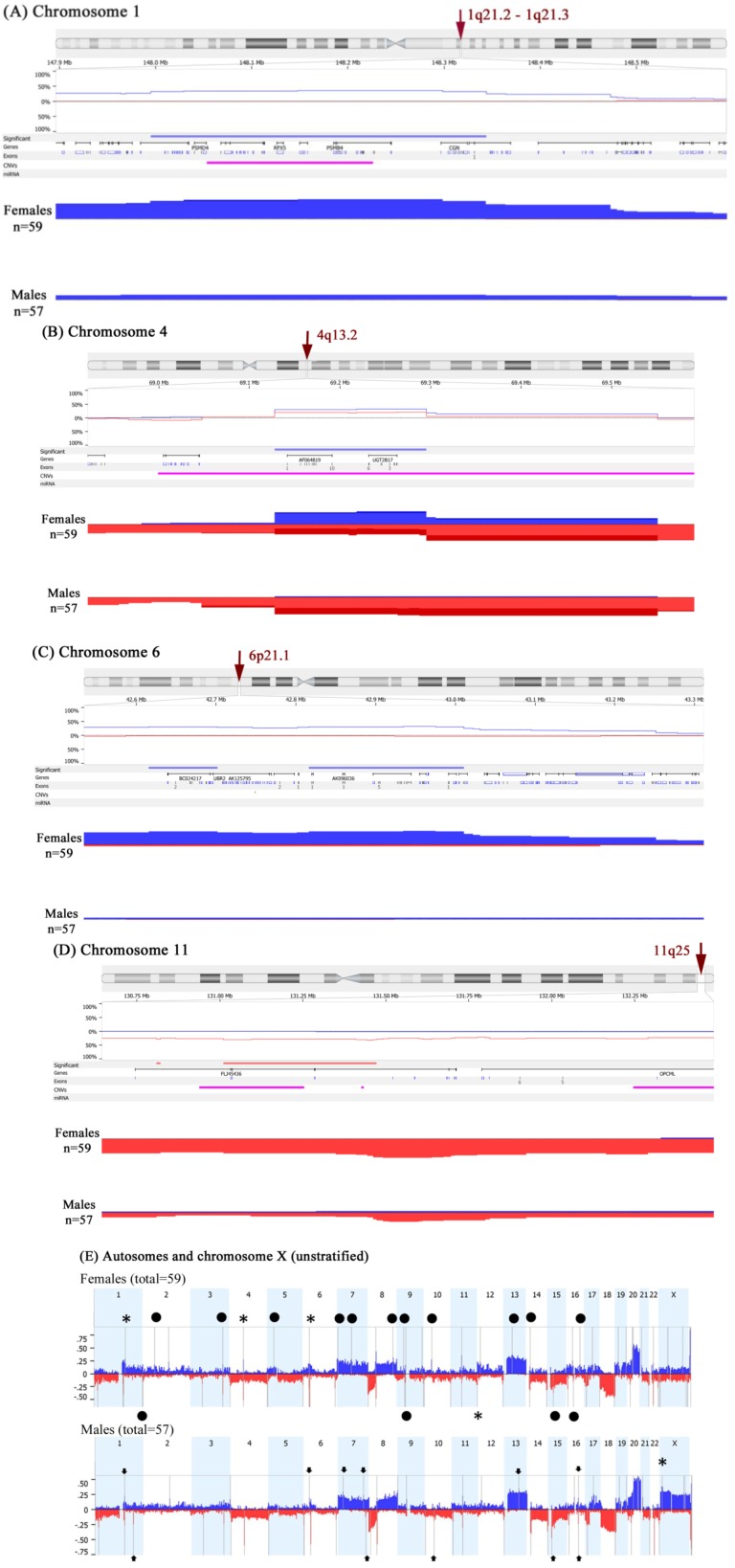
aCGH analysis of 116 colorectal cancers showing chromosomal copy number aberrations in male and female patients. Gains are depicted in blue and deletions in red. (**A**) 1q21.2–q21.3 locus; (**B**) 4q13.2 locus; (**C**) 6p21.1 locus; (**D**) 11q25 locus; (**E**) aCGH analysis using GISTIC algorithm showing copy number profiles of the autosomes and X-chromosome. The grey bars indicate highly significant aberrations as detected by GISTIC. Asterisks (*) indicate CNAs previously obtained by Significance Testing for Aberrant Copy number (STAC) then confirmed here. Black circles indicate statistically significant CNAs observed in females only, while arrowheads pinpoint CNAs specific to males.

Chromosomal CNAs were noted in male and female CRCs involving both autosomes and sex chromosomes. CRCs from female patients showed significantly more gains in chromosomal arms 1q21.2–q21.3, 4q13.2, 6p21.1, and 16p11.2, as well as copy number losses in chromosome arm 11q25 compared to male patients (Figure 1A–D and Table 2). This analysis was based on a global frequency statistical approach called Significance Testing for Aberrant Copy number (STAC) [15]. STAC-based algorithm is a robust method, which identifies a set of aberrations that are stacked on top of each other from different patients or microarrays such that it would not occur by chance. We further enhanced the analysis by using a second algorithm termed “GISTIC” for Genomic Identification of Significant Targets in Cancer, which identifies functionally significant CNAs by giving more weight to high copy gains and homozygous losses (amplitudes) that may be functionally relevant to the successful evolution of the cancer genome [16]. The GISTIC analysis confirmed the STAC determined CNAs (Figure 1E). The details of the GISTIC determined CNAs are presented in Supplementary File 1.

**Table 2:** Gender-specific copy number aberrations and associated genes in colorectal cancer.

Locus	CNA	Candidate Genes	Females %	Males %	*p*-Value
1q21.2	Gain	LINC00568, AF289612, AK023606, MIR4257, TSRC1, ADAMTSL4	50.8	17.5	<0.0001
1q21.2–1q21.3	Gain	U78576, PIP5K1A, BC007833, PSMD4, ZNF687, AK023105, PIK4CB, PI4KB, AJ011123, BC040300, RFX5, AK023875, SELENBP1, PSMB4, BX537561, POGZ, CGN, MIR554, TUFT1, AY358610	46.7	12.3	<0.0001
4q13.2	Gain	AF064819, UGT2B17	33.9	1.75	<0.0001
6p21.31	Gain	FKBP5, MIR5690	37.3	5.3	<0.0001
6p21.1	Gain	TBCC, AK096036, KIAA0240, GLTSCR1L, RPL7L1, C6orf226, LOC441150, PTCRA, CNPY3, TNRC5	35.6	3.5	<0.0001
11q25	Loss	FLJ45436, AY358331, NTM, BC050716, HNT	47.45	14	0.0001
16p11.2	Gain	QPRT, C16orf54, BC029149, ZG16, KIF22, BC004352, CR590954, AF489858, MAZ, L01420, BC041629, AK074572, PRRT2, LOC112476, AK092265, C16orf53, PAGR1, MVP, AK131349, CDIPT, LOC440356, BC000567, SEZ6L2, AJ245822, ASPHD1, LOC253982, KCTD13, TMEM219, LOC124446, TAOK2	54.2	20.8	0.0003

### 2.3. Feminization of Colorectal Cancer Cells in Males

**Figure 2:**
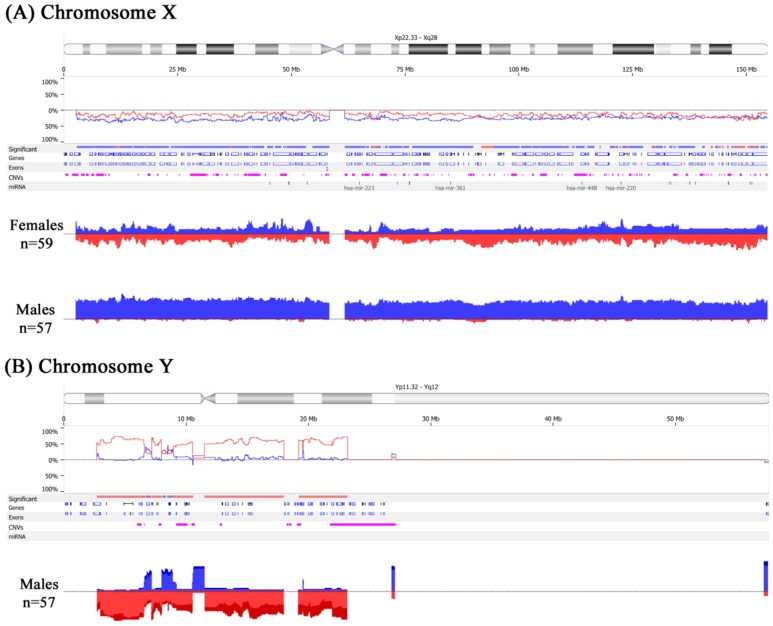
Sex-chromosome copy number aberrations in colorectal cancer cells by aCGH. Gains are depicted in blue and deletions in red. (**A**) Male cancers show extensive X chromosome gains while female cancers show equivalent frequencies of gains and losses; (**B**) Male cancers also show Y chromosome losses.

**Figure 3:**
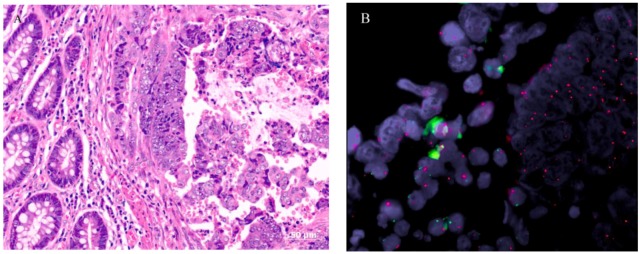
Morphology of a male colorectal cancer with feminization. (**A**) A hematoxylin and eosin stained histological section showing tumor on the right-hand side and normal colonic mucosa on the left; (**B**) Fluorescence *in-situ* hybridization of tumor cells (right-hand side) show multiple red centromeric enumeration probe (CEP) X signals indicating X chromosome gains and absence of green CEP Y signals indicating Y chromosome losses, while both red and green signals are present in non-neoplastic interstitial cells (left-hand side).

**Figure 4:**
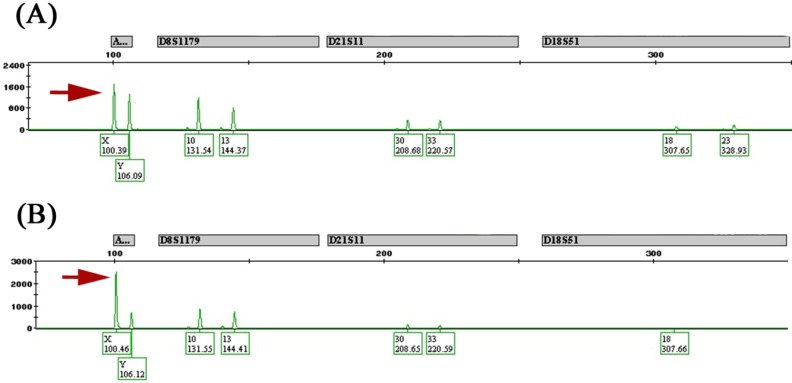
Allelotyping. (**A**) Normal DNA from a male patient showing X and Y peaks of equal height (arrow); (**B**) Tumor DNA from same patient showing X peak more than twice the height of the Y peak (arrow) indicating X gains.

Significant copy number differences of the sex chromosomes were observed in male patients, namely, extensive X chromosome copy number gains associated with Y chromosome losses at multiple loci (Figure 2A,B). We refer to this phenomenon as “feminization” and define it as any CRC arising in male patients showing gains of the X chromosome (or an arm of X chromosome) and/or losses of the Y chromosome. Table 3 shows the loci involved in the X chromosomal gains and Y losses that are of statistical significance along with the candidate genes that may be potentially involved by the CNA at those loci. Cancers arising in females, on the other hand, had equivalent frequencies of gains and losses of the X chromosome (Figure 2A). In total, 26 of 57 (46%) male CRCs showed feminization of cancer cells by aCGH. For confirmation of these results, a number of male and female cases were subjected to reflex testing by FISH and MLPA. Out of 15 male tumors tested by FISH, eight were feminized by aCGH and confirmed by FISH as follows: four showed increased centromeric enumeration probe (CEP) X signals and some loss of CEP Y signals in tumor cells (heterogeneous), three showed increased CEP X signals and complete loss of CEP Y signals in almost all cancer cells analyzed (homogeneous) (Figure 3A,B), and one tumor showed increased CEP X signals with preservation of CEP Y. The remaining seven of the 15 male tumors showed increased CEP X signals by FISH only. Of the 19 male samples tested by MLPA, 14 cancers showed concordant results with that of aCGH. Five feminized cancers by aCGH were not confirmed by MLPA (data not shown). Finally, as a quality control measure, eight tumor samples were subjected to allelotyping and were all confirmed to be from the correct corresponding individuals (*i.e.*, ruling out cross contamination between male and female samples) (Figure 4A,B).

**Table 3:** Copy number aberrations involving sex chromosomes in male and female colorectal cancers by array comparative genomic hybridization.

Locus	CNA	Candidate Genes	Females %	Males %	*p*-Value
Xp22.32–p22.31	Gain	NLGN4X, AK125309, MIR4770, VCX3A, HDHD1A, HDHD1, MIR4767, STS, VCX, PNPLA4, MIR651	9.3	42.3	<0.01
Xp22.31–p22.2	Gain	VCX-C, VCX3B, KAL1, FAM9A, AK097501, FAM9B, AY459291, TBL1X, GPR143, X83543, SHROOM2, APXL, CR749271, LOC100288814, KIAA1280, WWC3, BC035601, CLCN4, MID1, HCCS, AMELX, ARHGAP6, MSL3L1, MSL3, PDZK10, FRMPD4, PRPS2, TLR7, TLR8, AY358296, TLR8-AS1, TMSB4X, FAM9C, LOC100093698, ATXN3L, LOC100133123	10.7	45.8	<0.01
Xp22.12–p22.11	Gain	SH3KBP1, AL833278, CXorf23, AK094661, LOC729609, MIR23C, AK098768, MAP7D2, BC089400, FLJ14503, EIF1AX, SCARNA9L, RPS6KA3, CNKSR2, RP11-450P7.3, KLHL34, SMPX, BC036465, YY2, MBTPS2, SMS, PHEX, PHEX-AS1, ZNF645, LOC100873065, DDX53	12.5	46.2	<0.01
Xp22.11–p21.2	Gain	PDK3, BC045634, PCYT1B, SCARNA23, POLA, POLA1, ARX, MAGEB18, MGC33889, MAGEB6, MAGEB5, VENTXP1, SMEK3P, AK131475, AK057304, DCAF8L2, MAGEB10, DCAF8L1, IL1RAPL1, MAGEB2, MAGEB3, MAGEB4, MAGEB1, NR0B1	9.4	44.8	<0.01
Xp21.1–p11.4	Gain	DMD, MIR3915, BC036103, FAM47A, TMEM47, FAM47B, MAGEB16, CXorf22, RP13-11B7.1, CHDC2, CXorf30, FAM47C, LOC442444, AK125992, FTH1P18, PRRG1, AK130368, LANCL3, XK, CYBB, TCTE1L, DYNLT3, CXorf27, SYTL5, SRPX, RPGR, OTC, TSPAN7, TM4SF2, MID1IP1, LOC286442, AY316592, BCOR, ATP6AP2, BC010395, MPC1L, CXorf38, BC025334, AL832829, CRSP2, MED14	11.4	45.1	<0.01
Xq21.1–q21.31	Gain	APOOL, SATL1, ZNF711, ZNF6, BC067294, AK128541, AK026445, AK025039, POF1B, AF309774, BC017500, FLJ38564, CHM, BC032237, DACH2, AK022715, KLHL4, AB051474, LAMR1P15, CPXCR1	9.1	42.6	<0.01
Xq22.3	Gain	MID2, TEX13B, VSIG1, PSMD10, ATG4A, APG4A, AK054927, COL4A6	11.9	42.6	0.001
Xq13.1	Gain	EDA, MIR676, DGAT2L4, AWAT2, OTUD6A, HSHIN6, IGBP1	4.2	35.1	0.001
Xq22.1–q22.2	Gain	BEX4, BEXL1, TCEAL8, TCEAL5, BEX2, TCEAL7, WBP5	13.5	43.8	0.002
Xp11.22	Gain	LOC401589, SNORA11D, SNORA11E, AF329733, MAGED4, MAGED4B, XAGE2B, XAGE1A, XAGE1C, XAGE1D, XAGE1E, XAGE1, BC009538, XAGE1A, XAGE1B, XAGE2, XAGE1D, XAGE1B, SSX8, SSX7, SSX2, SSX2B, SPANXN5, SPANX-N5, XAGE5	8.5	39.6	0.004
Xq21.1	Gain	BX649166, HDX, CXorf43	11.2	41.6	0.008
Xp22.12	Gain	AK131412, MAP3K15	16.95	47.4	0.010
Xq22.1	Gain	RAB40AL, BEX1	10.2	40.4	0.010
Xq13.1	Gain	KIF4A, GDPD2, DLG3, AB033058	14.7	45.6	0.016
Xq22.3	Gain	MUM1L1, AK056478	8.5	38.6	0.019
Xq23	Gain	AMOT	10.2	40.4	0.020
Xq26.2	Gain	AL832725, PHF6, HPRT1	17.3	48.2	0.022
Xq22.3	Gain	AK001040, CXorf57, BC070110, FLJ10178, AK024253, RNF128	11.9	42.1	0.023
Xq23	Gain	SLC6A14	12.3	44.3	0.028
Xp11.23	Gain	WDR45, GPKOW, MAGIX, FLJ21687, PLP2, PRICKLE3, LMO6, SYP, SSX1	18.97	49.7	0.030
Xq22.2	Gain	RAB40A	11.4	42.3	0.030
Xq27.1	Gain	SRD5A1P1, F9	11.9	42.1	0.040
Xq28	Gain	AFF2, FMR2	11.9	42.1	0.042
Yp11.31–p11.2	Loss	SRY, RPS4Y1, ZFY, LINC00278, TGIF2LY, PCDH11Y, TTTY23, TTTY23B, TSPY1, TSPY2, TTTY1B, TTTY1, TTTY2B, TTTY2, TTTY21, TTTY7B, TTTY8B, TTTY8	–	67.89	<0.01
Yp11.2	Loss	PRKY, TTTY16, TTTY12	–	54.6	<0.01
Yq11.1–q11.221	Loss	GYG2P1, TTTY15, USP9Y, DDX3Y, UTY, BC071744, TMSB4Y, VCY, VCY1B, BC032567, NLGN4Y, NLGN4Y-AS1	–	57.4	<0.01
Yq11.222–q11.223	Loss	TTTY9A, HSFY2, NCRNA00185, CYorf14, CD24, TTTY14, BCORL2, BCORP1, CYorf15A, TXLNG2P, BC035312, CYorf15B, D87072, KDM5D, SMCY, TTTY10, EIF1AY, RPS4Y2, U88898, RBMY2EP, AK026367, RBMY1A1, BC070298, RBMY1F, RBMY1B, TTTY13, RBMY1D, RBMY1E, RBMY1A1, TTTY6, PRY, PRY2, AY597808, TTTY6B, RBMY1F, RBMY1J, RBMY1A1, BC047768, TTTY5, RBMY2FP, TTTY6	–	60.01	<0.01

**Figure 5:**
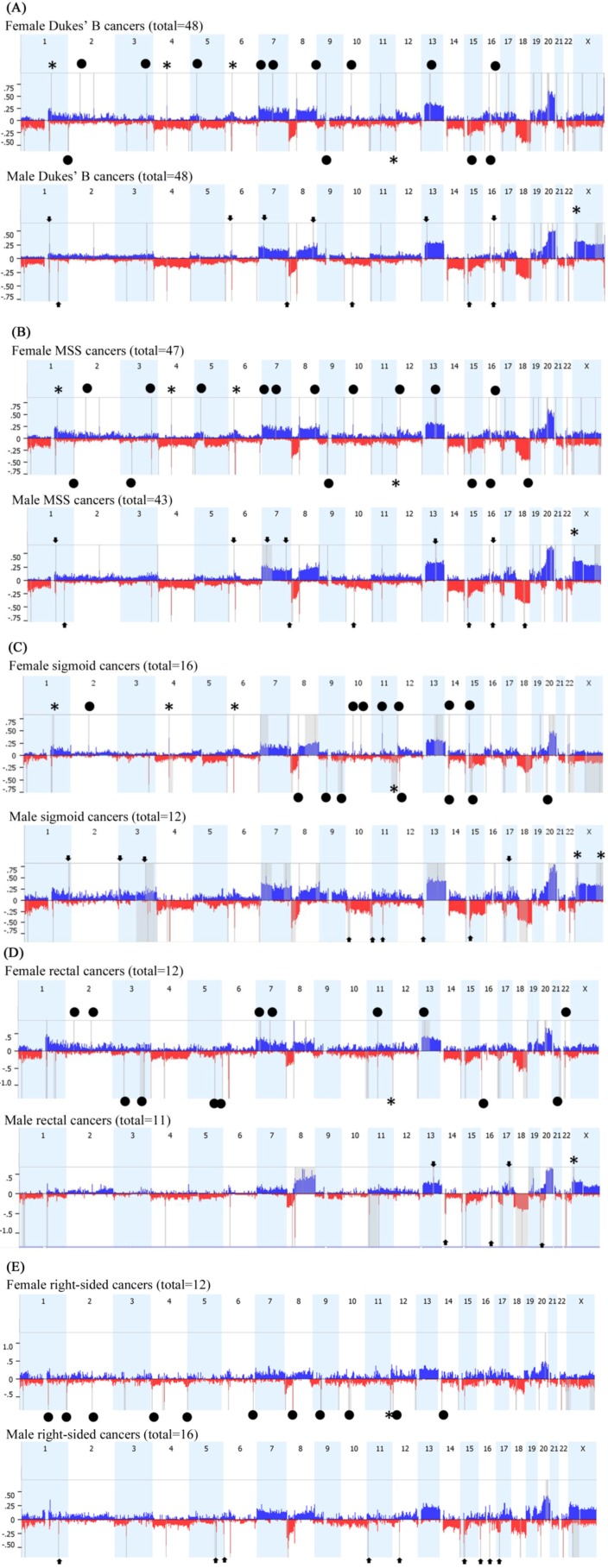
aCGH analysis of the autosomes and the X-chromosome using GISTIC algorithm limited to: (**A**) 96 cases of Dukes’ B cancer; (**B**) 90 cases of MSS cancer; (**C**) 28 cases with sigmoid colon cancer; (**D**) 23 cases with rectal cancer; (**E**) 28 cases with right-sided colon cancer. Gains are depicted in blue and deletions in red. The grey bars indicate highly significant aberrations as detected by GISTIC. Asterisks (*) indicate CNAs previously obtained by STAC then confirmed here. Black circles indicate statistically significant (*p* < 0.05) CNA specific to females, while arrowheads pinpoint CNA specific to males.

In order to minimize the potential of confounding factors, we have stratified the data by Dukes’ stage B (Figure 5A), microsatellite stable (MSS) status (Figure 5B), and cancer subsite for sigmoid colon (Figure 5C), rectum (Figure 5D) and right side of colon (Figure 5E). The resultant data were closely related to the unstratified GISTIC analysis presented in Figure 1E (Supplementary Files 2 and 3). Interestingly, CRC site appears to have a dramatic influence on gender-specific CNAs. While sigmoid colon cancers appear to display the same gender-specific CNAs seen in all CRCs overall, albeit with additional sigmoid-specific CNAs (Figure 5C and Supplementary File 4), rectal cancers appear to have only maintained the 11q25 loss in females and Xp22.32 gain in males (Figure 5D and Supplementary File 5). In right-sided colon cancer, gain of chromosome X in males was not significantly observed, and all other gender-specific CNAs were not detected except for loss of 11q25 in females, but other copy number losses were obvious between females and males (Figure 5E and Supplementary File 6).

### 2.4. Clinicopathological Significance

Statistically significant parameters in male “feminized” CRCs, were microsatellite stability (MSS) status and *BRAF* gene mutation status (Table 4). Of the 26 feminized male cancers, 24 (92%) tumors showed MSS/wild-type *BRAF* (*p*-value 0.003). This constitutes 58.5% of all MSS/wild-type *BRAF* in the male group. For *BRAF* status alone, all of the 26 feminized tumors showed a wild-type codon 600 of the *BRAF* gene (*p*-value 0.009), constituting 53% of all wild-type *BRAF* tumors in the male group. No significant differences were detected between feminized and non-feminized cases with respect to age, histological differentiation, Dukes’ stage, *KRAS* mutation status, metastatic relapse or disease-free survival.

**Table 4:** Clinicopathological characteristics of feminized colorectal cancers in male patients.

Characteristics	Feminized Male CRC (Total = 26) No. (%)	Non-Feminized Male CRC (Total = 31) No. (%)	Total Males (Total = 57) No. (%)	*p*-Value
MSS—wild type *BRAF*	24 (92.3)	17 (56.7)	41 (72.0)	0.0026 *
Wild-type *BRAF*	26 (100)	23 (74.2)	49 (86.0)	0.0088 *
MMR proficient	24 (92.3)	19 (61.2)	43 (75.4)	0.0107 *

* Statistically significant.

## 3. Discussion

Male and female CRCs demonstrate fundamental differences in several epidemiological, pathological, and clinical aspects. For example, men have a higher incidence of CRC than women overall [17,18]. With respect to cancer localization, left-sided CRC tends to be more frequent in men while right-sided CRC more frequent in women [19,20,21]. In our current study, however, left-sided tumors were more frequently seen in female patients (Table 1). CRCs demonstrating microsatellite instability (MSI) and CpG island methylator phenotype (CIMP-high) are significantly associated with the female gender, *BRAF* mutations and wild-type *KRAS* [22,23,24,25], whereas microsatellite-stable CRCs with less extensive promoter methylation (CIMP-low) are associated with the male gender and *KRAS* mutations [25,26]. Additionally, women with CRC seem to have an age-dependent survival advantage over men [18,27,28,29], with better survival seen in young to middle-aged women with localized disease [30,31] and worse survival in older women after menopause [32,33]. Some studies did not show a difference in survival between the two genders [34,35]. Sex hormonal influences and the protective effect of estrogen in women have long been proposed as a factor in this gender bias in the incidence and behavior of CRC [30,36], as opposed to testosterone, which is generally thought to contribute to increased aggressiveness of cancer in males [37,38]. Of note, the hormonal differences typical of the specific gender are unlikely to be seen in female patients older than 55 and male patients above 62 years of age. Nevertheless, the exact mechanisms underlying gender-related differences in CRC remain largely unexplained. Our study showed more cancer recurrences in female patients, however, there were no statistically significant differences in disease-free survival between men and women. This may be partly due to a selection bias of postmenopausal women in this study, since the majority of female patients were over the age of 50 years, or may be due to topographic differences in this region of the world. A recent study in Kuwait showed a mean recurrence-free survival time in CRC patients of 72.8 months and 65.2 months in males and females, respectively (*p*-value 0.025) [39].

We used high-resolution aCGH (244 K), a technique that screens the entire tumor genome for genetic gains and losses, in order to study chromosomal CNAs in CRC cells in relation to gender. Significant differences in CNAs between females and males were noted at several loci but the differences in the X and Y chromosome imbalances were particularly interesting. Twenty-six male CRCs, comprising 46% of all male cancers in this study, showed “feminization” in the form of extensive X chromosome gains with or without Y chromosome losses. Our aCGH results were further confirmed by multiplex ligation-dependent probe amplification (MLPA) and fluorescence *in situ* hybridization (FISH). In cases subjected to MLPA, 73% showed strong correlation with aCGH data, while those subjected to FISH 57% showed strong correlation with aCGH data. Five of the feminized male cancers identified by aCGH were not confirmed by MLPA. This discrepancy between aCGH and MLPA is expected and can be explained by the fact that the MLPA technique detects a small gene locus on the X chromosome that may not be involved in the CNA, and therefore MLPA offers a more limited view of the X chromosome.

The gender-specific wide-genome CNAs reported here might have been influenced by several confounding factors, although in this cohort female and male CRCs did not differ significantly in terms of clinicopathological characteristics (Table 1). For this reason, we have stratified our CNA analysis to take into account the potential influences of Dukes’ stage, microsatellite status and cancer subsite. After stratification, the gender-specific aberrations were confirmed, but interestingly our data indicate that these gender-specific CNAs may be site-specific. For example, while sigmoid colon cancers in males appear to display significant feminization, right-sided colon cancer did not demonstrate significant gains of the X chromosome. This intriguing observation deserves attention and confirmation in larger cohorts.

X chromosome gains have previously been observed in a variety of tumors including hepatoblastoma [40], prostate cancer [41], and testicular germ cell tumors [42], amongst others. With respect to CRC, Dutrillaux *et al*. [11,12] first described the gain of early-replicating (active) X chromosomes in male and female CRCs, demonstrating that the X gains were associated with a frequent loss of either the late-replicating (inactive) X chromosome in females or the Y chromosome in males. De Angelis *et al.* [6] demonstrated a high level of X chromosome gains by CGH in a series of 45 CRCs with a higher percentage of Xq gain in male patients and X losses in females. Brim *et al.* [14] also demonstrated the preferential gain of chromosome X in male CRC cells in a study of 30 CRCs from African American male patients, a high-risk group for CRC. The hypothesis postulated is that increased “dosage” of expressed X-linked genes may play a key role in oncogenesis [6,11,43] and that the higher incidence of CRC in males may be related to the lack of X-chromosomal inactivation [13]. In normal female cells, random inactivation of one of the X chromosomes occurs early in life making female somatic tissues mosaic with respect to whether the maternal or paternal X chromosome is active [43]. Therefore, gain of X chromosomes in male cancer cells may increase the expression of oncogenes carried on the X chromosome that are related to CRC [13].

Similar to the results of Unotoro *et al.* [13], the current study shows significant gains of the Xp21 locus in male CRC cells (*p*-value < 0.01), which is the location of the *MAGE-B* gene family (melanoma-associated antigen gene) [44]. Another significantly gained locus was Xp11.22 (*p*-value 0.004), which hosts the *XAGE* genes as well as the *SSX* genes (human synovial sarcoma on X chromosome) that are rearranged in synovial sarcoma. *MAGE*, *SSX*, and *XAGE* genes all belong to the cancer-testis antigen family of genes that are normally expressed in the testis and aberrantly expressed in a wide array of human cancers [45,46]. *MAGE* and *SSX* co-expression has been recently correlated with liver metastasis in colorectal cancer [47]. At the present time, however, it is difficult to know, which genes are of significance in feminized CRCs due to the large genomic areas affected by the X chromosomal aberrations. With respect to the Y chromosome losses in male CRC observed here, other studies also support our findings [11,12,48].

Other than sex chromosome CNAs, tumors from females showed significantly more gains at loci 1q, 4q, 6p and 16p compared to males and significantly more losses at 11q. Gains of 1q and 4q have previously been demonstrated in CRC [49] but were not correlated to gender. Several other chromosomal gains and losses have also been described in CRC, for example gains of 3q, 7p, 8q, 13q, 17p, 18q, 20q [4,6,7,9] and losses of 1p, 4, 5q, 18q and 17p [5,7,8,9]. However, the relationship between these CNAs and gender has rarely been studied. What kind of influence these gender-associated CNAs have on CRC needs further exploration in larger cohorts.

In the present study, feminized CRCs correlated with MSS and wild-type *BRAF* status. Conversely, tumors that lacked feminization were mostly MSI tumors, *BRAF*-mutated, and right-sided. MSS is an expected finding in feminized CRCs, as MSI and chromosomal instability (feminization is a form of chromosomal instability) are less likely to coexist in the same tumor [50,51]. However, more recent evidence suggests that MSI and chromosomal instability may not be mutually exclusive [52,53,54]. Since MSS CRCs may have a worse prognosis than MSI cancers [55,56,57,58], we have restricted our disease-free survival analysis to the “MSS group” of patients and found that males with and without feminized tumors did not differ in disease-free survival rate (data not shown). No significant correlation was found between feminization and age, histological differentiation, Dukes’ stage, *KRAS* mutation status or disease-free survival. Larger studies are required to confirm these results.

In summary, differences in chromosomal CNAs between genders may be an important aspect in the development of CRC, and feminization may constitute a unique pathway in colorectal carcinogenesis in a subset of male patients, which warrants further investigation using larger cohorts.

## 4. Materials and Methods

### 4.1. Patients and Tissue Samples

This study was approved by the Ethical Committee (2006-1302-07, 13 April 2008) of the Faculty of Medicine at Kuwait University. A cohort of 116 patients with CRC was used for genetic analysis. Formalin-fixed paraffin-embedded (FFPE) tissue samples were retrieved from pathology archives and clinical information (gender, age, tumor site in the colon, tumor histological grade, Dukes’ stage, and clinical outcome data) was retrieved from patients’ charts.

### 4.2. Array Comparative Genomic Hybridization (aCGH)

Our standard published protocol was used to process the FFPE tissue samples for aCGH [59]. Briefly, 2 μg of tumor DNA and pooled sex-matched reference DNA (Promega, Fitchburg, WI, USA) were fragmented in a water-bath sonicator (Elmasonic, Singen, Germany), then labeled using the Cy3 and Cy5 universal linkage system dyes (Agilent Technologies, Santa Clara, CA, USA). The labeled DNA was purified using the Agilent-KREApure columns (Agilent Technologies), and hybridized on to Human 244A slides (Agilent Technologies). Hybridization was carried out in SureHyb Chambers (Agilent Technologies) for 40 h at 60 °C. Slides were then washed and scanned at 5 μm resolution using an Agilent microarray scanner. Scanned images were imported, and the background was subtracted and normalized using Feature Extraction Software (Agilent Technologies). The feature extraction software generates a Quality Control Report, which determines the quality of the aCGH. Quality Control metrics such as Derivative of Log Ratio Spread (DLRSpread), Back Ground Noise, Signal Intensity, Reproducibility and Signal to Noise ratio are generated in the QC report.

DLRSpread is defined as the spread of the Log Ratio differences between consecutive probes along all chromosomes. It enhances the ability to measure the noise of the log ratio independently of the number and severity of aberrations found. Samples with DLRSpread higher than 0.5 were excluded from further analysis. The text files representing data ratio points log2 of test/control ratios were imported to Nexus software (Biodiscovery, El Segundo, CA, USA). Quality values ranged between 0.05 and 0.4, which are excellent values given the degraded nature of the samples. To minimize false positive calls and random copy number variations, Fast Adaptive State Segmentation Technique (FASST2) with a stringent significance threshold of 5.0 × 10^−6^ was used to determine CNAs for each sample. CNA comparisons between the two groups (Males *vs.* Females) were performed within Nexus using the Fisher’s exact test. The Q-bound value, which corrects for multiple testing by performing False Discovery Rate (FDR) correction, was used to highlight significant CNA differences between the two groups.

### 4.3. Fluorescence in Situ Hybridization (FISH)

To confirm the aCGH results, sex-chromosome copy number counts by FISH was performed in 30 tumor samples (15 males and 15 females). For that we used the CEP X SpectrumOrange/Y SpectrumGreen DNA Probe Kit (Abbott Molecular, North Chicago, IL, USA), which detects alpha satellite sequences in the centromere region of the X chromosome (Xp11.1–q11.1) and satellite III DNA at the Yq12 region of the Y chromosome. We performed this 2-color FISH on 3-μm-thick tissue sections, which were then counterstained using 4'6-diamidino-2-phenylindole (DAPI). The Capturing and counting of the signals were performed using the “Metafer” system (Metasystems, Newton, MA, USA) on a Carl–Zeiss fluorescent microscope (Zeiss, Oberkochen, Germany).

### 4.4. Multiplex Ligation-Dependent Probe Amplification (MLPA)

For further verification, 44 tumor samples (19 males, 25 females) were subjected to MLPA to assess the gender of cancer cells. The MLPA assay was performed using the SALSA^®^ MLPA^®^ probemix P294-A1 tumor-loss kit (MRC-Holland; Amsterdam, The Netherlands). The hybridization, ligation, and amplification steps were performed according to the manufacturer’s standard protocol and the data analysis was done using the Coffalyser NET software created by MRC-Holland, Amsterdam, The Netherlands. The data generated from CRC samples were normalized and quality checked against reference controls. MLPA result reports, including descriptive statistics, ratios, 95% confidence intervals, and predictions, were exported to Microsoft Excel software data sheets for further analysis.

### 4.5. Allelotyping

As a quality control measure, randomly selected eight-paired normal and corresponding tumor tissues were subjected to allelotyping in order to rule-out cross contamination between male and female tissue samples. The identities of tumor tissues and their corresponding normal tissues were validated using PowerPlex-16 HS System (Promega, Fitchburg, WI, USA) according to the manufacturer’s instruction manual. Genomic DNA was extracted using the Qiagen DNA extraction kit (producer, Hilden, Germany). Detection of amplified fragments was performed using Life Technologies 3130xl Genetic Analyzer and GeneMapper software version 4.0 (Foster City, CA, USA).

### 4.6. Clinicopathological Correlation and Statistical Analysis

Chromosomal CNA data generated from aCGH were correlated with clinicopathological parameters including tumor location within the colon, histopathological differentiation, Dukes’ stage, microsatellite instability (MSI) status, mismatch repair (MMR) gene function, *BRAF* gene mutation status (p.V600E), and *KRAS* gene status (pre-determined using the standard Sanger sequencing method). Statistical analysis was calculated using Chi-square or Fisher’s exact tests and *p*-values of <0.05 were considered statistically significant. Disease-free survivals were quantified by the generation of Kaplan–Meier curves, and detection of cancer recurrence in the form of metastasis or local recurrence was used as an endpoint measurement. Patients who did not relapse (“endpoint”) during the study period (1990–2009) but died of other reasons or were lost to follow-up were censored. The mean period of follow-up was 7.5 years.

## 5. Conclusions

Feminization of colorectal cancer cells in male patients warrants further investigation using larger cohorts.

## Supplementary Materials

Supplementary materials can be found at http://www.mdpi.com/1422-0067/15/10/17344/s1.

## Supplementary Files

Supplementary File 1

## References

[1] Markowitz S.D., Bertagnolli M.M. (2009). Molecular origins of cancer: Molecular basis of colorectal cancer. N. Engl. J. Med..

[2] Ried T., Knutzen R., Steinbeck R., Blegen H., Schröck E., Heselmeyer K., du Manoir S., Auer G. (1996). Comparative genomic hybridization reveals a specific pattern of chromosomal gains and losses during the genesis of colorectal tumors. Genes Chromosomes Cancer.

[3] Bomme L., Bardi G., Pandis N., Fenger C., Kronborg O., Heim S. (1994). Clonal karyotypic abnormalities in colorectal adenomas: Clues to the early genetic events in the adenoma-carcinoma sequence. Genes Chromosomes Cancer.

[4] Bardi G., Sukhikh T., Pandis N., Fenger C., Kronborg O., Heim S. (1995). Karyotypic characterization of colorectal adenocarcinomas. Genes Chromosomes Cancer.

[5] Al-Mulla F., Keith W.N., Pickford I.R., Going J.J., Birnie G.D. (1999). Comparative genomic hybridization analysis of primary colorectal carcinomas and their synchronous metastases. Genes Chromosomes Cancer.

[6] De Angelis P.M., Clausen O.P., Schjolberg A., Stokke T. (1999). Chromosomal gains and losses in primary colorectal carcinomas detected by CGH and their associations with tumour DNA ploidy, genotypes and phenotypes. Br. J. Cancer.

[7] He Q.J., Zeng W.F., Sham J.S., Xie D., Yang X.W., Lin H.L., Zhan W.H., Lin F., Zeng S.D., Nie D. (2003). Recurrent genetic alterations in 26 colorectal carcinomas and 21 adenomas from Chinese patients. Cancer Genet. Cytogenet..

[8] Al-Mulla F., Behbehani A.I., Bitar M.S., Varadharaj G., Going J.J. (2006). Genetic profiling of stage I and II colorectal cancer may predict metastatic relapse. Mod. Pathol..

[9] Tsafrir D., Bacolod M., Selvanayagam Z., Tsafrir I., Shia J., Zeng Z., Liu H., Krier C., Stengel R.F., Barany F. (2006). Relationship of gene expression and chromosomal abnormalities in colorectal cancer. Cancer Res..

[10] Dorak M.T., Karpuzoglu E. (2012). Gender differences in cancer susceptibility: An inadequately addressed issue. Front. Genet..

[11] Dutrillaux B., Muleris M., Seureau M.G. (1986). Imbalance of sex chromosomes, with gain of early-replicating X, in human solid tumors. Int. J. Cancer.

[12] Muleris M., Dutrillaux A.M., Salmon R.J., Dutrillaux B. (1990). Sex chromosomes in a series of 79 colorectal cancers: Replication pattern, numerical, and structural changes. Genes Chromosomes Cancer.

[13] Unotoro J., Kamiyama H., Ishido Y., Yaginuma Y., Kasamaki S., Sakamoto K., Oota A., Ishibashi Y., Kamano T. (2006). Analysis of the relationship between sex and chromosomal aberrations in colorectal cancer by comparative genomic hybridization. J. Int. Med. Res..

[14] Brim H., Lee E., Abu-Asab M.S., Chaouchi M., Razjouyan H., Namin H., Goel A., Schäffer A.A., Ashktorab H. (2012). Genomic aberrations in an African American colorectal cancer cohort reveals a MSI-specific profile and chromosome X amplification in male patients. PLoS One.

[15] Diskin S.J., Eck T., Greshock J., Mosse Y.P., Naylor T., Stoeckert C.J., Weber B.L., Maris J.M., Grant G.R. (2006). STAC: A method for testing the significance of DNA copy number aberrations across multiple array-CGH experiments. Genome Res..

[16] Beroukhim R., Getz G., Nghiemphu L., Barretina J., Hsueh T., Linhart D., Vivanco I., Lee J.C., Huang J.H., Alexander S. (2007). Assessing the significance of chromosomal aberrations in cancer: Methodology and application to glioma. Proc. Natl. Acad. Sci. USA.

[17] Rim S.H., Seeff L., Ahmed F., King J.B., Coughlin S.S. (2009). Colorectal cancer incidence in the United States, 1999–2004: An updated analysis of data from the National Program of Cancer Registries and the Surveillance, Epidemiology, and End Results Program. Cancer.

[18] Purim O., Gordon N., Brenner B. (2013). Cancer of the colon and rectum: Potential effects of sex–age interactions on incidence and outcome. Med. Sci. Monit..

[19] DeCosse J.J., Ngoi S.S., Jacobson J.S., Cennerazzo W.J. (1993). Gender and colorectal cancer. Eur. J. Cancer Prev..

[20] McCashland T.M., Brand R., Lyden E., de Garmo P., Project C.R. (2001). Gender differences in colorectal polyps and tumors. Am. J. Gastroenterol..

[21] Takada H., Ohsawa T., Iwamoto S., Yoshida R., Nakano M., Imada S., Yoshioka K., Okuno M., Masuya Y., Hasegawa K. (2002). Changing site distribution of colorectal cancer in Japan. Dis. Colon Rectum.

[22] Ward R., Meagher A., Tomlinson I., O’Connor T., Norrie M., Wu R., Hawkins N. (2001). Microsatellite instability and the clinicopathological features of sporadic colorectal cancer. Gut.

[23] Hawkins N., Norrie M., Cheong K., Mokany E., Ku S.L., Meagher A., O’Connor T., Ward R. (2002). CpG island methylation in sporadic colorectal cancers and its relationship to microsatellite instability. Gastroenterology.

[24] Ogino S., Cantor M., Kawasaki T., Brahmandam M., Kirkner G.J., Weisenberger D.J., Campan M., Laird P.W., Loda M., Fuchs C.S. (2006). CpG island methylator phenotype (CIMP) of colorectal cancer is best characterised by quantitative DNA methylation analysis and prospective cohort studies. Gut.

[25] Ogino S., Goel A. (2008). Molecular classification and correlates in colorectal cancer. J. Mol. Diagn..

[26] Ogino S., Kawasaki T., Kirkner G.J., Loda M., Fuchs C.S. (2006). CpG island methylator phenotype-low (CIMP-low) in colorectal cancer: Possible associations with male sex and KRAS mutations. J. Mol. Diagn..

[27] Elsaleh H., Joseph D., Grieu F., Zeps N., Spry N., Iacopetta B. (2000). Association of tumour site and sex with survival benefit from adjuvant chemotherapy in colorectal cancer. Lancet.

[28] Kotake K., Honjo S., Sugihara K., Kato T., Kodaira S., Takahashi T., Yasutomi M., Muto T., Koyama Y. (2003). Changes in colorectal cancer during a 20-year period: An extended report from the multi-institutional registry of large bowel cancer, Japan. Dis. Colon Rectum.

[29] Micheli A., Ciampichini R., Oberaigner W., Ciccolallo L., de Vries E., Izarzugaza I., Zambon P., Gatta G., de Angelis R. (2009). EUROCARE Working Group. The advantage of women in cancer survival: An analysis of EUROCARE-4 data. Eur. J. Cancer.

[30] Hendifar A., Yang D., Lenz F., Lurje G., Pohl A., Lenz C., Ning Y., Zhang W., Lenz H.J. (2009). Gender disparities in metastatic colorectal cancer survival. Clin. Cancer Res..

[31] Majek O., Gondos A., Jansen L., Emrich K., Holleczek B., Katalinic A., Nennecke A., Eberle A., Brenner H. (2013). GEKID Cancer Survival Working Group. Sex differences in colorectal cancer survival: Population-based analysis of 164,996 colorectal cancer patients in Germany. PLoS One.

[32] Koo J.H., Jalaludin B., Wong S.K., Kneebone A., Connor S.J., Leong R.W. (2008). Improved survival in young women with colorectal cancer. Am. J. Gastroenterol..

[33] Koo J.H., Leong R.W. (2010). Sex differences in epidemiological, clinical and pathological characteristics of colorectal cancer. J. Gastroenterol. Hepatol..

[34] Ponz de Leon M., Sant M., Micheli A., Sacchetti C., di Gregorio C., Fante R., Zanghieri G., Melotti G., Gatta G. (1992). Clinical and pathologic prognostic indicators in colorectal cancer. A population-based study. Cancer.

[35] Manfredi S., Bouvier A.M., Lepage C., Hatem C., Dancourt V., Faivre J. (2006). Incidence and patterns of recurrence after resection for cure of colonic cancer in a well defined population. Br. J. Surg..

[36] Slattery M.L., Potter J.D., Curtin K., Edwards S., Ma K.N., Anderson K., Schaffer D., Samowitz W.S. (2001). Estrogens reduce and withdrawal of estrogens increase risk of microsatellite instability-positive colon cancer. Cancer Res..

[37] Shahabi S., He S., Kopf M., Mariani M., Petrini J., Scambia G., Ferlini C. (2013). Free testosterone drives cancer aggressiveness: Evidence from US population studies. PLoS One.

[38] Orsted D.D., Nordestgaard B.G., Bojesen S.E. (2014). Plasma testosterone in the general population, cancer prognosis and cancer risk: A prospective cohort study. Ann. Oncol..

[39] Al-Qallaf D.A., Al-Mulla F. (2012). Colorectal Carcinoma in Kuwait: Apoptosis and Its Relation to Clinicopathological Characteristics, p53 Expression and ki-ras Proto-Oncogene Mutations. Ph.D. Thesis.

[40] Terracciano L.M., Bernasconi B., Ruck P., Stallmach T., Briner J., Sauter G., Moch H., Vecchione R., Pollice L., Pettinato G. (2003). Comparative genomic hybridization analysis of hepatoblastoma reveals high frequency of X-chromosome gains and similarities between epithelial and stromal components. Hum. Pathol..

[41] Visakorpi T., Hyytinen E., Kallioniemi A., Isola J., Kallioniemi O.P. (1994). Sensitive detection of chromosome copy number aberrations in prostate cancer by fluorescence *in situ* hybridization. Am. J. Pathol..

[42] Looijenga L.H., Oosterhuis J.W. (1999). Pathogenesis of testicular germ cell tumours. Rev. Reprod..

[43] Spatz A., Borg C., Feunteun J. (2004). X-chromosome genetics and human cancer. Nat. Rev. Cancer.

[44] Muscatelli F., Walker A.P., de Plaen E., Stafford A.N., Monaco A.P. (1995). Isolation and characterization of a *MAGE* gene family in the Xp21.3 region. Proc. Natl. Acad. Sci. USA.

[45] Dos Santos N.R., Torensma R., de Vries T.J., Schreurs M.W., de Bruijn D.R., Kater-Baats E., Ruiter D.J., Adema G.J., van Muijen G.N., van Kessel A.G. (2000). Heterogeneous expression of the SSX cancer/testis antigens in human melanoma lesions and cell lines. Cancer Res..

[46] Simpson A.J., Caballero O.L., Jungbluth A., Chen Y.T., Old L.J. (2005). Cancer/testis antigens, gametogenesis and cancer. Nat. Rev. Cancer.

[47] Choi J., Chang H. (2012). The expression of *MAGE* and *SSX*, and correlation of COX2, VEGF, and survivin in colorectal cancer. Anticancer Res..

[48] Bottarelli L., Azzoni C., Necchi F., Lagrasta C., Tamburini E., D’Adda T., Pizzi S., Sarli L., Rindi G., Bordi C. (2007). Sex chromosome alterations associate with tumor progression in sporadic colorectal carcinomas. Clin. Cancer Res..

[49] Xiao X.Y., Zhou X.Y., Yan G., Sun M.H., Du X. (2007). Chromosomal alteration in Chinese sporadic colorectal carcinomas detected by comparative genomic hybridization. Diagn. Mol. Pathol..

[50] Lothe R.A., Peltomäki P., Meling G.I., Aaltonen L.A., Nyström-Lahti M., Pylkkänen L., Heimdal K., Andersen T.I., Møller P., Rognum T.O. (1993). Genomic instability in colorectal cancer: Relationship to clinicopathological variables and family history. Cancer Res..

[51] Muleris M., Dutrillaux A.M., Olschwang S., Salmon R.J., Dutrillaux B. (1995). Predominance of normal karyotype in colorectal tumors from hereditary non-polyposis colorectal cancer patients. Genes Chromosomes Cancer.

[52] Goel A., Arnold C.N., Niedzwiecki D., Chang D.K., Ricciardiello L., Carethers J.M., Dowell J.M., Wasserman L., Compton C., Mayer R.J. (2003). Characterization of sporadic colon cancer by patterns of genomic instability. Cancer Res..

[53] Camps J., Armengol G., del Rey J., Lozano J.J., Vauhkonen H., Prat E., Egozcue J., Sumoy L., Knuutila S., Miró R. (2006). Genome-wide differences between microsatellite stable and unstable colorectal tumors. Carcinogenesis.

[54] Trautmann K., Terdiman J.P., French A.J., Roydasgupta R., Sein N., Kakar S., Fridlyand J., Snijders A.M., Albertson D.G., Thibodeau S.N. (2006). Chromosomal instability in microsatellite-unstable and stable colon cancer. Clin. Cancer Res..

[55] Hawkins N.J., Tomlinson I., Meagher A., Ward R.L. (2001). Microsatellite-stable diploid carcinoma: A biologically distinct and aggressive subset of sporadic colorectal cancer. Br. J. Cancer.

[56] Sinicrope F.A., Rego R.L., Halling K.C., Foster N., Sargent D.J., La Plant B., French A.J., Laurie J.A., Goldberg R.M., Thibodeau S.N. (2006). Prognostic impact of microsatellite instability and DNA ploidy in human colon carcinoma patients. Gastroenterology.

[57] Kazama Y., Watanabe T., Kanazawa T., Tanaka J., Tanaka T., Nagawa H. (2007). Microsatellite instability in poorly differentiated adenocarcinomas of the colon and rectum: Relationship to clinicopathological features. J. Clin. Pathol..

[58] Watanabe T., Kobunai T., Yamamoto Y., Matsuda K., Ishihara S., Nozawa K., Yamada H., Hayama T., Inoue E., Tamura J. (2012). Chromosomal instability (CIN) phenotype, CIN high or CIN low, predicts survival for colorectal cancer. J. Clin. Oncol..

[59] Al-Mulla F. (2011). Microarray-based CGH and copy number analysis of FFPE samples. Methods Mol. Biol..

